# Emerging Roles of Complement Protein C1q in Neurodegeneration

**DOI:** 10.14336/AD.2019.0118

**Published:** 2019-06-01

**Authors:** Kyoungjoo Cho

**Affiliations:** Department of Life Science, Kyonggi University, Suwon, South Korea

**Keywords:** innate immunity, C1q, aging, neurodegenerative disease, synaptic pruning, neuron, astrocyte, microglia

## Abstract

The innate immune system is an ancient and primary component system that rapidly reacts to defend the body against external pathogens. C1 is the initial responder of classical pathway of the innate immune system. C1 is comprised of C1q, C1r, and C1s. Among them, C1q is known to interact with diverse ligands, which can perform various functions in physiological and pathophysiological conditions. Because C1q participates in the clearance of pathogens, its interaction with novel receptors is expected to facilitate apoptosis induction, which could prevent the onset or progression of neurodegenerative diseases and could delay the aging process. Because senescence-associated secreting phenotype determinants are generally inflammatory cytokines or immune factors to activate immune cells. In the central nervous system, C1q has diverse neuroprotective roles against pathogens and inflammation. Most of neurodegenerative diseases show region specific pathology feature in the brain. It has been suggested the evidences that the active site and amount of C1q may be disease specific. This review considers currently the emerging and under-recognized roles of C1q in neurodegeneration and highlights the need for further research to clarify these roles. Future studies on the roles of C1q in regulating disease progression should consider these aspects, including the age-dependent onset time of each neurodegenerative disease progression.

Review

## 1. Introduction

The defense system against damage in the body involves innate and adaptive immunity. The innate immune system is an ancient and primary component system designed to rapidly detect and react against attacks by external pathogens [1]. The innate immune response is performed by a complement system that contains three complement pathways: the classical pathway, lectin pathway, and alternative pathway. This response involves soluble complement proteins, which make up approximately 5% of the total protein content of human blood plasma [2]. In the classical pathway, the C1 complement protein is the initial responder of the classical pathway and is comprised of C1q, C1r, and C1s [3]. In the presence of calcium, C1q, C1r, and C1s can form a complex that induces conformational changes in the collagen region of C1q to activate the classical pathway.

C1q is actually involved in various cellular functions of the classical complement pathway, including cellular differentiation [4], intercellular adhesion [5], chemotaxis [3], aggregation of cellular macromolecules [5], pathogenesis of neurodegenerative diseases [6], and clearance of apoptotic cell debris [7]. C1q also has anti-cancer effects via immune surveillance and may participate in the aging process [8]. C1q is the subcomponent that recognizes several ligands in the classical complement pathway [5] and alters acceptor molecules [9], thus C1q play a bridge role between innate and adaptive immunity.

C1q, which modulates the immune responses of a variety of cells, is also produced by cells of the central nervous system (CNS). In the CNS, C1q plays a role in synaptic pruning during CNS development process [10]. C1q also has a broad neuroprotective role during the inflammatory response to pathogens. However, C1q has also deleterious interaction with abnormal protein aggregates and involves in the progression of neurodegenerative diseases. Innate immunity has been currently emerging as an important factor of neurodegenerative disease including cognitive disorder. Recent decades, neuroinflammation has been pointed as a new and important condition of Alzheimer’s disease. Despite, the roles of C1q in another neurodegenerative diseases are still under-recognized. This review provides that complement factor C1q has a role in neurodegenerative disease pathology and discuss how to be involved in developing diseases. Furthermore, this bolsters the need for further investigations of C1q in aging.

## 2. General form and function of C1q

In mammals, the complement system is central in innate and adaptive immunity and is activated through three pathways based on the primary components to recognize pathogens. The first is the classical complement pathway, which is initially and primarily recognized by C1q. The second is the lectin pathway, which is sensed by mannose-binding protein. The third is the alternative pathway, which is primarily detected by C3 [5]. C1q acts as a recognition molecule in the classical complement pathway, performing both complement and non-complement functions.

### 2.1 Structural form of C1q

**Figure 1.**
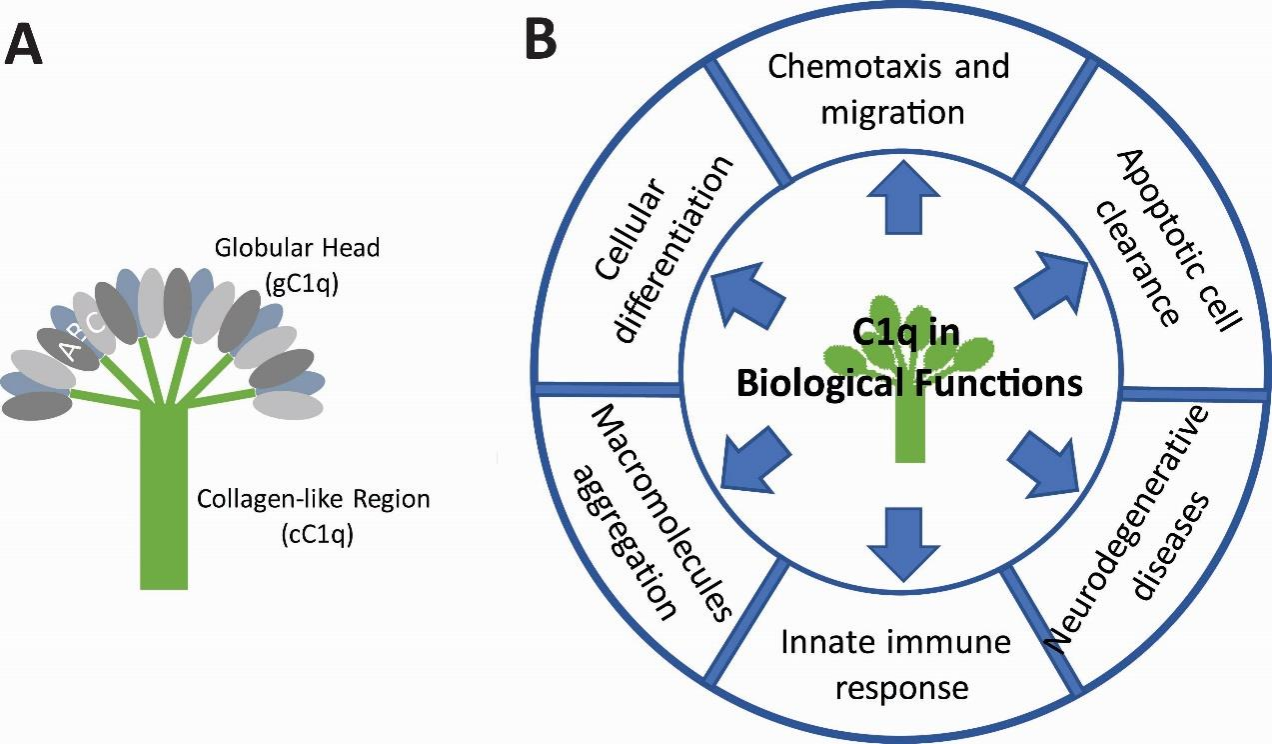
Structural form and biological functions of C1q (**A**) C1q consists of globular heads (gC1q) and a collagen-like region (cC1q). (**B**) The diagrammatic circle is presenting the functional hallmark of C1q.

C1 is a calcium-dependent trimolecular complex made up of C1q, C1r, and C1s, which are assembled in a 1:1:1 ratio. C1q is primarily synthesized in tissue macrophages of myeloid cells [11]. In healthy humans, the soluble proteins of the complement system are approximately 5% of the total protein contents in the human blood plasma [12]. The C1q concentration in normal human serum ranges from 56~276 μg/ml. Non-circulating C1q is restricted in peripheral tissues and is about 10% of the total C1 protein complex. Among the three subunits, free circulating C1q represents approximately 10% of the total physiological concentration [12]. The human C1q molecule weighs 460 kDa and consists of 18 polypeptides with up to 220 residues each. The structure produces two unique structural and functional domains: One domain contains a short flanking N-terminal region termed the collagen-like region (CLR or cC1q), and the other is a globular C1q domain (gC1q) presenting at the C-terminus [13, 14]. These two C1q domains are independent of each other and interact with various other biological structures, including pathogen- and cell-associated molecules (Fig. 1A).

C1q consists of the six trimeric globular heads made of three polypeptide chains, A, B, and C. Each chain has its own ligand and each ligand specifically recognizes different molecular counterparts (or partners) [15, 16]. The globular heads are assembled with a structure of six collagen-like stalks, which appear to be fibril-like and exist in the central region. The cC1q collagen-like sequences form a triple-helical collagen-like structural unit in the chains, resulting in an ABC-CBA arrangement [17]. These subunits are held together with covalent and non-covalent bonds, and three central units are associated via strong non-covalent bonds. The final structure of the C1q molecule resembles a bouquet of tulip bulbs [18]. This structure initiates the classical complement pathway and the consequent formation of the membrane attack complex (MAC). C1q is not a lectin, however, it is considered (/called) as a member of lectin (collectin / ficolins). Because C1q has similar features functionally as well as structurally with mammalian lectins and ficolins. They are oligomeric proteins and they have also carbohydrate-recognition domains: C-type lectin carbohydrate-recognition domain (lectin) or fibrinogen like carbohydrate-recognition domains (ficolins). Likewise, C1q contains carbohydrate-recognition domain for danger-associated molecular patterns (DAMPs) or pathogen-associated molecular patterns (PAMPs). With these features, C1q anchors on the membrane and serves as one of pathogen recognition receptor (PRR) by recognizing damage molecules with globular head domains of C1q [15, 16]. It could be that the collectins and the C1q protein involves in the inflammation or specifically in adaptive immune response. Furthermore, C1q is able to accelerate apoptotic cell clearing by macrophages, which is the point to involve the disease pathology or progression.

### 2.2 Biological function of C1q

In the early stages of infection or disease, only a marginal adaptive immune response to the pathogen occurs. Instead, C1q facilitates a crucial mechanism through the innate immune system [3] by enhancing the phagocytosis of opsonized pathogens in which collagen-immobilized surfaces bind directly to the target molecules [19, 20]. Once C1q binds its specific targeting ligands, the classical pathway is activated in IgG-dependent and -independent manners. Membrane-associated C1q can sense the patterns of danger by capturing globular head domains [5]. The recognition of PAMPs or DAMPs modulates the inflammatory response leading cytokines secretion, chemokines release, apoptosis, phagocytosis, and angiogenesis [21]. Tumor necrosis factor-alpha (TNF-α), one of the triggers of the immune response, is involved in the function of C1q [22, 23]. When C1q binds to the cell surface along with C1qR, C1q-mediated chemotaxis enhanced by extracellular gC1qR occurs. In the cytosol, activated C1q receptors result in the increased content of calcium ions and hyperpolarization of potassium channels, which can preclude the migration of fibroblasts to C1q [24]. Consistent with this, another study reported that C1q facilitates adhesion between fibroblasts and extracellular matrix material, such as collagen and fibronectin [25].

C1q is locally synthesized and induces a variety of biological functions by autocrine or paracrine signaling molecules. The available evidence supports the “local-synthesis-for-local function” paradigm, in which C1q is synthesized in a specific locus functioning only at that locus [17]. In addition to this localized function, the findings of in vitro chemotaxis assays indicate that C1q concentration may affect the migration of mast cells to the sites of the inflammatory response [26]. When cells are injured or encounter trauma, the concentration of free C1q becomes so high that chemotaxis of neutrophils can be stimulated, resulting in their movement to the chemotactic lesion at the site of injury [27]. The migration of human neural stem cells has also been described [28]. The chemotaxis role of C1q may also involve lung epithelial permeability. In this scenario, neutrophils are recruited to the sites of cellular events, accumulating proteins such as C1q, IgM, and albumin [29].

Besides the involvement of C1q in the classical pathway, C1q appears to have additional roles in homeostasis and cellular development [30]. C1q has also been implicated in superoxide (O_2_-) production by neutrophils [31], blood coagulation [32], pregnancy [33], and development, particularly in neurological synapse pruning [34]. The various biological functions of C1q are depicted in Figure 1B. As stated above, the suggested features of C1q and several lines of evidence raise the possibility that C1q may play a role in neurological pathologies of the CNS. This is considered in detail in the next section.

## 3. The roles of C1q in the central nervous system

Generally, the functions of the classical complement pathway have been elucidated in the peripheral system, whereas its functions in the central nervous system (CNS) remain unclear. Recent studies have indicated the functions of the classical complement pathway during normal brain development and aging process [10, 35-37]. C1q and major histocompatibility complex (MHC) I colocalize in developing synapses of the dorsolateral geniculate nucleus (dLGN). It means that complement proteins participate in the neuronal developing stage, but not in the immune pathway [38]. However, it remains unclear how these complement components interact to shape the brain and establish neural circuits according to development.

### 3.1 Development: synaptic pruning

A redundant and spider web-like neural network grows during CNS development, leading to excessive synaptic formation and potential synaptic malfunction [39]. The mammalian newborn brain contains excess neuronal connections (synapses). As the connections mature, precise neural circuits become more important. Therefore, during development, the brain must actively remove excess connections. This process is termed synaptic pruning [40-42]. Complement components are critical in the developmental process of synaptic pruning. This process removes less active or “weak” synapses and strengthens the appropriate connections, resulting in neuronal maturation [10]. This process is different from neuronal activity. Among complement components, the C1q, C3, and C4 complement factors participate in synapse elimination by tagging on abnormal synapses or inappropriate synaptic connections. Once these neurons are marked by complement factors, they are removed by phagocytic microglia via synaptic pruning process. The failure of this refinements in neuronal connectivity could lead to neurological disorders [43].

Experimental data have shown the failure of synaptic pruning in mice deficient in C1q, C3, or C4 [44, 45]. The tagging of synapses by C3 is dependent on the upstream complement components, C1q and C4. By this synergic line of the classical complement cascade, unnecessarily branched synapses can be eliminated to produce a mature CNS circuit. In a study involving a CNS mouse model, C1q was upregulated with peak production two weeks after birth [44]. During normal development, complement proteins of the brain are produced locally because immune cells are blocked by the blood-brain barrier, which acts to protect the CNS from macromolecules and plasma-derived macrophagic cells [46].

The retinogeniculate system is a classic experimental model for synaptic refinement [44, 47-49]. In the developing visual system, C1q is expressed only during the developmental phase. In early postnatal mice, retinal ganglion cells (RGCs) of each eye are arranged on the same side of neurons in the dLGN of the thalamus, and eye-specific territories are consequently co-localized [50-52]. However, in the retinal neurons of adult mice, RGCs do not display co-localized eye-specific territories. This arrangement is consistent with the suggestion that eye-specific territories result from extra-synaptic connections and the removal of excess synapses during synaptic pruning. Fluorescent anterograde tracers can be visualized by intraocular injection. Using cholera toxin beta to label RGC axons, the retinal pruning process is demonstrated [44, 53]. In RGC axons terminals in the dLGN, they observed that the C3 protein was localized for tagging and that TGF-β induced C1q [54]. These results indicated that C1q is essential for normal synaptic refinement in the developing visual system.

In *Xenopus laevis*, C1q and other compliment factors are expressed in embryos. These factors participate differently according to embryo patterning or organ development [55]. C1qA is dominantly expressed in the gastrula mesoderm and becomes mesodermal derivatives (vascular tissues and pronephros). Correspondingly, the C1qR expression is detected in the neural plate of the neural crest and in the developing vasculature. Because neural crest cells migrate and develop into several types of cells, C1qA, C3, C3aR, C9, and properdin are expressed in these cells. This provides insight into the contribution of receptor ligand interaction to neural crest cell migration and differentiation [55]. Later in development, C3a expression is evident in the endoderm and, reciprocally, C3aR is detected in the visceral mesoderm. Additionally, intertissue communication with complement factors containing C1q has been suggested to participate in Xenopus gut development. This scenario is supported by the finding that the mesodermal transcription factor FoxF1 helps modulate gut development in mice [55, 56].

In the developmental stage, the failure of synaptic pruning is an essential aspect of epileptogenesis [57]. Epilepsy is a prominent example of neuropathogenesis. For example, in C1q knock-out mice unable to establish proper synaptic connectivity, spontaneous epileptiform activity was observed [57]. The absence of synaptic pruning can lead to lengthened dendrites, increased branching, and augmented density of dendritic spines [58]. Several neurodevelopmental disorders are thought to be caused by a failure or imbalance in synaptic pruning, sparking interest in elucidating the role of complement proteins including C1q during the neurodevelopmental stage.

### 3.2 Aging

Similar to the development stage, C1q is differentially expressed and involved in aging, another time-dependent process. Recent evidence described the changing level of C1q in mouse CNS synapse that matched the evidence found in zebrafish or *Xenopus laevis* embryogenesis [59]. With age, C1q protein expression increases up to 300-fold, mainly secreted from microglia and some neurons [35]. Some studies showed that immunoreactive C1q-positive signals were detected in microglia of the postnatal brain, and co-localized with GABAergic neurons in the hippocampal dentate gyrus [60]. Additionally, the dramatic increases of C1q protein have been detected in the brains of normally aging mice and humans, particularly in the pyriform cortex, substantia nigra, and hippocampus [36].

**Figure 2.**
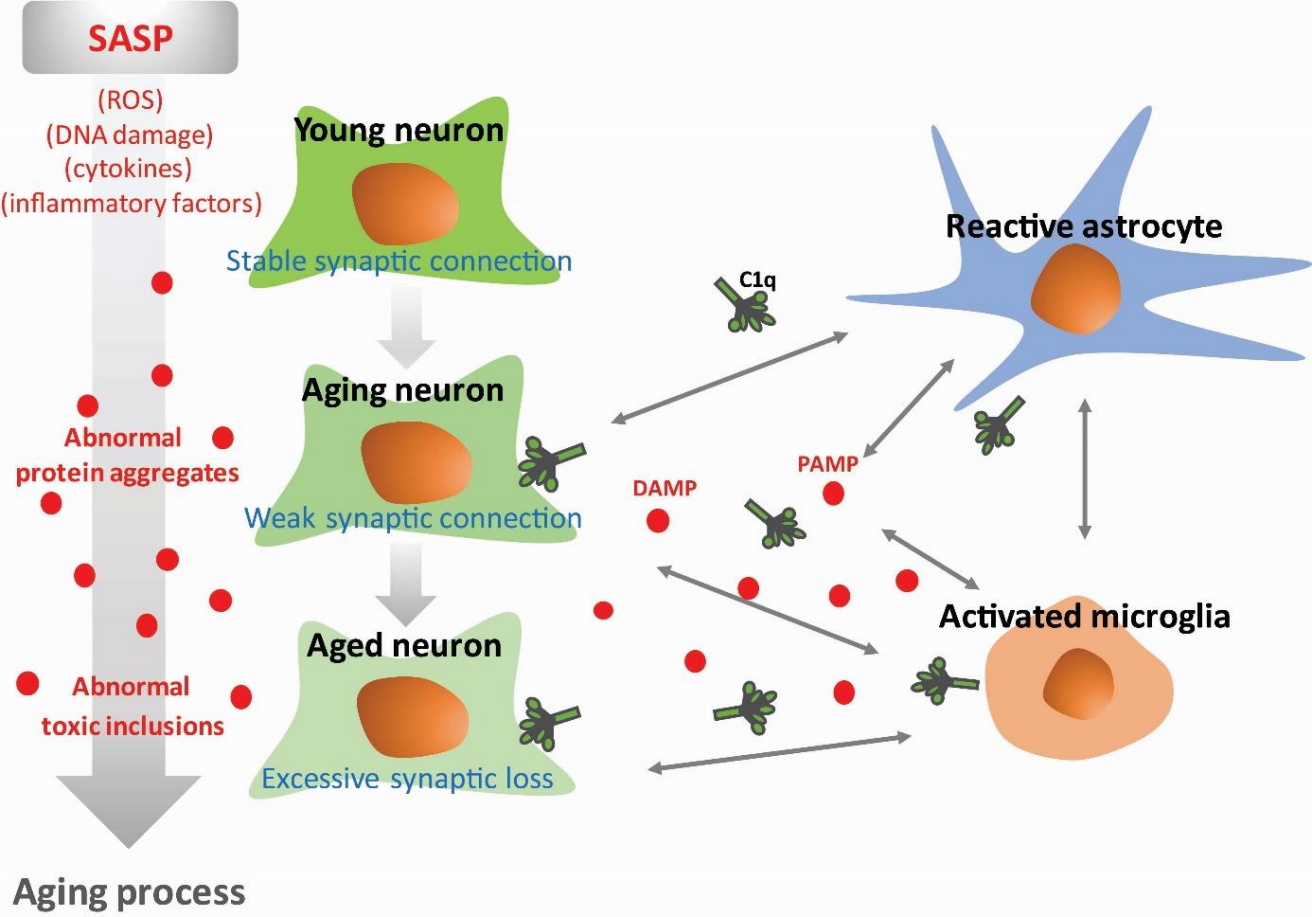
C1q in the CNS aging process Systemic changes due to aging occur as aged neurons are degenerated by the activation of surveillance microglia. Increased C1q that accompanies aging factors, but not the increased amount itself, may contribute to the aging progression of neurons. CNS-derived C1q is thought to correspond to physiological changes known as senescence-associated secreting phenotype determinants, which are relevant to ROS, DNA damages, and inflammatory cytokines. DAMP, danger-associated molecular pattern; PAMP, pathogen-associated molecular pattern.

During CNS maturation, the C1q protein was reported to be substantially increased and affect dentate gyrus-dependent synaptic plasticity in adults. It was different pattern between C1q knock-out (KO) mice and wild-type (WT), although behavioral dysfunction was not displayed in middle-aged (3-month-old) KO and WT mice [36]. The study reported that C1q did not participate in the classical pathway via the C3 component and did not affect synapse loss at least until middle-age stage. However, unlike in the developing or middle-age CNS, aged C1q-KO mice displayed cognitive decline, including memory function and some hippocampus-relevant behavior tests [36]. These results indicate that increased C1q that accompanies aging, but not the increased amount itself, may contribute to the progression of cognitive decline with aging (Fig. 2).

**Figure 3.**
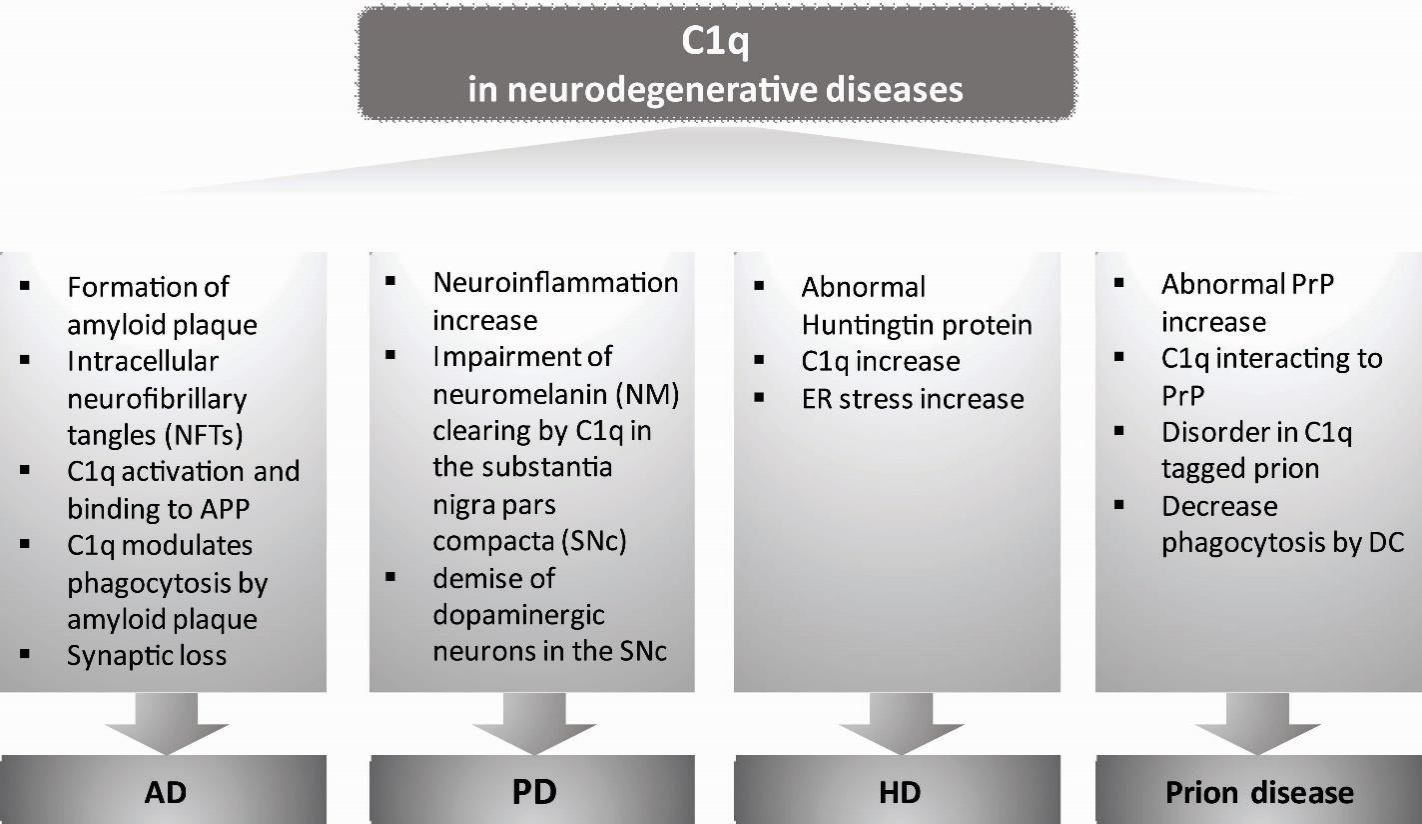
Pathophysiological implications of C1q in neurodegenerative diseases. C1q is involved in the pathological pathway in each neurodegenerative disease due to its role in abnormal protein aggregate clearance, astrocyte reactivation, binding and activation of microglia, or inflammatory responses. C1q in the age-dependent onset time of each neurodegenerative disease participates in regulating disease progression. AD, Alzheimer’s disease; PD, Parkinson’s disease; HD, Huntington’s disease; PrP, prion protein; DC, dendritic cell.

Generally, CNS-derived C1q has been thought to correspond to physiological changes. However, a study indicated that C1q-KO aged mice did not show any functional abnormalities of hippocampus in physiological condition [61]. This evidence also supported the existence of another age-related factor affecting cognitive decline, rather than aging-related cognitive dysfunction that leads to the malfunction of developmental processes. Additionally, determinants of the senescence-associated secreting phenotype (SASP) are relevant to inflammatory cytokines and immune cell activation. Therefore, it is important to explore how C1q participates in regulating neurodegenerative disease progression in the context of the age-dependent onset time of each disease. Nevertheless, it seems to be true that a higher level of C1q in the aging brain correlates with cognitive deficits. Several lines of evidences show that synaptopathy or synaptic dysfunction is an early feature of the neurodegeneration in diseases, such as Alzheimer’s disease (AD), Parkinson’s disease (PD), and Huntington’s disease (HD) (Fig. 3) [62, 63]. The roles of C1q in each neurodegenerative disease are discussed in another section.

## 4. The roles of C1q in each CNS cell type

In conditions of inflammation, external injury, and cellular stress in the CNS, complement proteins are expressed and secreted from neurons, microglia, and astrocytes [64]. Each cell takes a part in CNS and plays a role in synapse formation, homeostasis, macrophagic phagocytosis, and scar formation. Microglia and other cell types of brain are activated responding to misfold protein aggregates found in various neurodegenerative diseases.

### 4.1. Microglia

Microglia in the CNS act as part of the surveillance of the surrounding area and as specialized macrophages [65]. Rapidly responding to damage, microglia morphologically transform into phagocytic macrophages that secrete cytokines, chemokines, and nitric oxide (NO). Activated microglia with an adequate MHC expression profile migrate to the site of injury. In synaptic pruning, microglia-derived C1q with a role may be an essential source of synapse removal via the complement system. Under normal conditions, this protein is maintained at a low level. However, once microglia are activated, the C1q level increases to promote pro-inflammatory cytokines, such as interleukin-6 (IL-6) and TNF-α, and results in neuronal death [66, 67]. In a study of human microglial cells, fibril formation of prion protein (PrP) was increased when sufficient amounts of C1q were present [68]. Thus, an increase of C1q in active microglia could lead to the secretion of pro-inflammatory IL-6 and TNF-α [68]. An increased C1q transiently raises the level of reactive oxygen species (ROS), NO, and calcium and can also halt microglial proliferation [66]. Enhanced phagocytosis by microglia is induced by the binding of C1q to apoptotic cells or neuronal blebs [66]. When microglia were activated by C1q in vivo, lipopolysaccharide-induced TNF-α and IL-6 levels decreases [69]. These results indicate a specific response mechanism.

Very recently, the role of microglia in CNS diseases has been elucidated, particularly, as a regulating partner rather than just an executioner of phagocytosis that is known its classic function in the CNS. In line with the above-mentioned studies, C1q derived from microglia involves in neurodegenerative disease and may modulate the neuropathologic condition.

### 4.2. Astrocytes

The second cell type in the CNS is astrocytes. It has been reported that astrocytes produce C1q in response to cerebral fungal infection [70]. Astrogliosis is a scar formation process. In astrogliosis, the morphological change and reactivation of astrocytes produce a scar in response to CNS damage; this scar formation is associated with C1q and cytokine secretion [71, 72]. For example, in patients with multiple sclerosis, detected CNS plaques are significant signs that C1q is co-localized with reactive astrocytes [73]. Recently, astrocyte-derived TGF-β was found to induce C1q in purified RGCs [54]. Disruption of the cytokine signaling pathway can result in TGF-β inhibition of C1q-dependent synaptic pruning [54]. The results are described that C1q can regulate microglia-mediated synaptic pruning. Several recent studies have indicated that astrocyte-derived C1q may modulate neuronal synapse weakening and degradation during neurodegeneration [6, 8]. Among CNS insults, traumatic injury or ischemic condition can also fully activate complement system. In this pathologic condition, astrocytes are also reactive and trigger complement factors including C1q [68].

### 4.3. Neurons

The final major cell type in the CNS is neurons. In addition to microglia and astrocytes, neurons also produce C1q [74]. The secretion level of neuron-derived C1q can depend on the immature astrocytes that secrete cytokines like thrombospondins [44]. In a study using C1q- or C3-null homozygous animals, the contralateral optic fibers did not segregate with the ipsilateral fibers, an abnormal event that lasted 30 days following birth. During this time, most of the LGN neurons were innervated with many synapse inputs, and the amplitudes of post-synaptic currents were shown to be responsive to the amplitudes of pre-synaptic current stimulation [44]. Other studies showed that hippocampal neurons secrete C1q [75] to protect neuronal cells from β-amyloid-induced damage under β-amyloid burden [76]. The level of C1q expression in neurons is also related to cholesterol levels because the alteration of C1q expression induces neurite outgrowth due to lowered cholesterol in neurons [77]. C1q is involved in upregulating the cholesterol-25-hydroxylase gene, whose expression is associated with cholesterol distribution or lipid metabolism [77]. C1q also plays a role in upregulating nerve growth factor (NGF) by increasing its transcription factors or decreasing microRNA-targeting NGF [77].

The complement factor C1q derived from CNS cells is also associated with neuroprotection against external infections, such as meningitis [78]. C1q is considered to be beneficial for eliminating aggregated proteins following the activation of the complement factor by low levels of aggregates. However, when the complement factor is chronically activated, it can harm the CNS due to activated microglia and pro-inflammatory cytokines [79, 80]. In the CNS, many components of the immune system are locally produced. These proteins are involved in maintaining homeostasis similar to the function of the peripheral system [64].

Several neurodevelopmental disorders are thought to be caused by a failure or imbalance in synaptic pruning. It is the results in the combined working of neuron, microglia, and astrocytes. It is sparking interest in elucidating the role of complement proteins including C1q during the neurodevelopmental stage. In a dysregulated complement system that promotes imbalanced synaptic pruning, C1q could act as a trigger of neurodegenerative diseases in their early stages and stimulate synapse loss in the developing and mature brain [10]. Collectively, it can be summarized that complement components including C1q have beneficial and detrimental roles in the CNS. This knowledge could lead to the development of therapeutic interventions for neuroinflammation and neurodegenerative diseases.

## 5. Emerging importance of C1q in neurodegenerative diseases

Apart from the role of C1q in CNS infections, a novel role for C1q has been newly uncovered in the neuropathological pathways implicated in traumatic brain injury, neurodegenerative diseases, and even psychiatric disorders. Furthermore, C1q has been implicated in prion disease progression, synaptic pruning in CNS development, and aging [81]. Although synaptic loss resulting from developmental synaptic pruning has not been confirmed as a leading cause of these detrimental conditions, complement components are suggested as therapeutic targets for neuroprotection or for delaying the progress of neurodegenerative diseases.

### 5.1. C1q in Alzheimer’s disease

Many studies have indicated that C1q and the classical complement pathway are involved in age-related neuronal diseases that feature massive synapse loss and cognitive deficits [5, 42]. Among them, C1q can interact with amyloid precursor protein (APP) and APP metabolites that are regulated according to developmental stages. In a study involving postnatal hamsters, RGCs bound to APP and the complex were selectively detected in the sites of axon elongation or synapse formation [82].

Generally, soluble APP (sAPP) acts as a brain neurotrophic factor associated with several synapse proteins including N-methyl-D-aspartic acid receptor. These proteins participate in synaptic plasticity, long-term potential, and spatial memory [83]. The other form, the soluble extracellular N-terminal peptide of APP (NAPP), is a pro-apoptotic ligand for the TNF family member Death Receptor 6 (DR6) [83]. When NAPP is activated, the apoptotic pathway is initiated for the selective elimination of excessive neurons or synapses in neuronal dendrites [81, 84]. C1q modulates the process of phagocytosis by microglia responding to amyloid plaque (Aβ peptides). However, the associations of the fibrillar form of Aβ in AD and C1q expression and other inflammatory factors remain unclear. Another process still waiting to be clarified is the formation of extended amyloid plaque during the disease progression. By contrast, the pathological form is a soluble fibrillar form, which hinders neuronal plasticity by inhibiting synaptic vesicle release through the exchange of calcium currents on the presynapse [85].

Various roles have been assigned to C1q in diverse diseases, even in the reverse consequences according to diseases or disease stages. Therefore, it is hard to decide whether a higher level of C1q is beneficial or harmful. For a clinical approach, C1q has to be examined in the whole systemic context. The brain is a relatively isolated region from the whole body; this will make it effective to confer the roles of C1q and to elucidate its changed level in neurological disease onset and progression.

### 5.2 C1q in Parkinson’s disease

Another neurodegenerative disease is Parkinson’s disease (PD), which is clinically characterized by the akinetic-rigid syndrome. The major feature of PD is a progressive demise of dopaminergic neurons in the substantia nigra pars compacta (SNc) [86]. Although compliment cascades and complement factors have been suggested to contribute to neurodegenerative diseases, few studies have assessed the association between the complement pathway and PD.

A study of autopsy samples from patients with PD and control subjects clarified the neuroinflammatory processes in PD [87]. Several studies identified neuroinflammation in the brains of patients with PD using functional brain scanning and PK-11195, a microglial benzodiazepine receptor ligand [88, 89]. Activated microglia have been observed in animal models of PD, and it has been suggested that the modification of innate immune response plays a role in neuronal protection [90, 91]. Although microglia-derived C1q have been detected in the brains of healthy individuals and those with PD, C1q levels are upregulated in the SNc of the brains from patients with PD. Furthermore, C1q was shown to remove neuromelanin and other neuronal cell debris in the SNc subregion. The time-dependent movement of monocyte-derived cells from the brain to the perivascular region was also reported [92, 93]. C1q-positive cells were found in neuromelanin located in the outer region of the blood-brain barrier in the SNc of brains from PD patients [87]. Until this report, C1q had not been detected by immunostaining of Lewy bodies and dendritic spheroid bodies, in contrast to the success of identifying C3d, C4d, C7, and C9 by immunostaining [94].

### 5.3 C1q in Huntington’s disease

Huntington’s disease (HD) is characterized to affect selectively medium spiny GABAergic neurons (MSNs) in the striatum. Among MSNs, specific kinds of neurons expressing D2-dopamine receptor, neurotensin, or metenkephalin are particularly vulnerable [95]. Also, HD is one of neurodegenerative disease by age-dependent disease onset. It has been related to immune response including C1q expression in the striatum [96, 97]. Recently, several results have been suggested that inflammatory responses are contributing in disease pathology with post-mortem study [98]. In the brain of the patients with HD, astrogliosis and microgliosis are upregulated. The affected striatal region of the brain showed the increased level of microglia-derived complement factors and also IL-1β. According to proteomic analysis of HD patients’ plasma, several complements factors are detected as well as TNF levels were elevated in the early stages of HD patients. It was corresponding to the increased mRNA levels from the HD patients’ brain such as CCL2, IL10, IL6, IL8 and TNF [99]. Furtherly the relationship of immune factors between plasma concentration and mRNA level in the brain was correlated with disease progression. Likewise, IL-6 also increased in patients with HD, and became more abundant as the disease progressed [100]. Actually, C1q binds to apoptotic cells and then enhances microglia to secrete TNF and IL6 for phagocytosis [66].

On the contrary, another study reported that increased IL-6 levels in the plasma was already detected before the onset of any disease symptoms in carriers with HD [101]. In the R6/2 mouse model, proinflammatory genes including TNF, interferon-γ (IFNγ) and TGFβ1 was significantly upregulated in the symptomatic striatal tissue [102]. It was correlated and co-localized with C1q expression level. They may as potential key regulators of HD symptom onset and clinical progression.

Generally, HD studies have focused on the pathology relevant to mutant huntingtin protein (mHtt) and the prevention against mHtt synthesis, aggregation, and inclusion. Several studies elucidated that mHtt may induce innate immune activation both in the brain and peripheral tissues. It is linked that complement factors including C1q and symptomatic progression. Now, it is needed to explain and explore how innate immune pathways work with HD, which will serve as future therapeutic targets for HD.

## 6. Perspectives and conclusion

C1q has an important role in health and disease as a classic participant in innate immunity. Its role in the fields of neurodegenerative diseases and cognitive dysfunction has been emerging. The characteristics of C1q and its potential therapeutic importance can be summarized as follows: First, C1q is an early responder to pathologic conditions and rapidly reacts with the pathologic changes; therefore, the C1q level could be an effective diagnostic or prognostic marker for neurodegenerative disease onset and progression. Second, C1q recognizes diverse ligands and makes them proceed to the next step including adaptive Immune response, being a potential target for pharmaceutical application.

The role of C1q in the neurodegenerative pathophysiology, as well as neuronal development, needs to be clarified. Its function in the aging process including neurodegenerative disease needs to be determined to better understand diseases with neuronal involvement. C1q has been highlighted in complement system research for the last decade. In physiological and pathophysiological conditions, the localized expression of C1q in both immune and non-immune cells works in different ways to maintain the homeostatic machinery according to receptors. Because C1q takes part in the clearance of pathogens and apoptotic cells, C1q’s interaction with novel receptors could help inducing apoptosis, preventing the onset or progression of neurodegenerative diseases, and delaying the aging process. The location and amount of C1q may be disease specific. Therefore, it is important to explore how C1q participates in regulating age-dependent onset time and symptomatic progression of each neurodegenerative disease in the context of the aging process
